# Epidemiological survey of adult female stress urinary incontinence

**DOI:** 10.1186/s12905-021-01319-z

**Published:** 2021-04-22

**Authors:** Rui Qin Zhang, Man Cheng Xia, Fan Cui, Jia Wei Chen, Xiao Dong Bian, Hong Jie Xie, Wei Bing Shuang

**Affiliations:** 1 Department of First Clinical Medical College, Shanxi Medical University, Taiyuan, 030001 Shanxi China; 2 Department of Urology, The First Affiliated Hospital of Shanxi Medical University, Taiyuan, 030001 Shanxi China

**Keywords:** Stress urinary incontinence, Epidemiology, Risk factor

## Abstract

**Background:**

The prevalence of stress urinary incontinence (SUI) in adult female in Taiyuan and what are the related risk factors are not clear. The aim of this study was to provide a basis for exploring the prevention and treatment of SUI in adult female in Taiyuan.

**Methods:**

A voluntary online questionnaire was used to investigate adult female in the community and surrounding townships of Taiyuan. Most of the questionnaires refer to the International Consultation on Incontinence Questionnaire-Female Lower Urinary Tract Symptoms, and adapt to the specific circumstances of the region. Data were analyzed using SPSS software (version 22.0).

**Results:**

A total of 4004 eligible questionnaires were obtained. The prevalence of SUI in adult female in Taiyuan was 33.5%. Univariate analysis and multivariate logistic regression analysis showed that place of residence, smoking, body mass index, diet, number of deliveries, mode of delivery, dystocia, menopause, oral contraceptives, urinary tract infection, making the bladder empty faster by pushing down and holding urine were risk factors for adult female stress urinary incontinence in Taiyuan.

**Conclusion:**

The prevalence of SUI in adult female in Taiyuan was high, and based on risk factors identified in this survey, population-level intervention strategies should be developed for the prevention and treatment of adult female SUI in Taiyuan.

**Supplementary Information:**

The online version contains supplementary material available at 10.1186/s12905-021-01319-z.

## Background

Stress urinary incontinence (SUI) is defined as “the complaint of involuntary loss of urine on effort or physical exertion (e.g., sporting activities), or on sneezing or coughing” [1], and it is the most common type of urinary incontinence. Although SUI does not threaten the patient's life, it seriously affects the patient's quality of life [2–4]. In recent years, the prevalence of female SUI has gradually attracted public attention. Epidemiological surveys of regional populations have been conducted in many regions in China [5–7], while there is still a lack of relevant epidemiological data in Taiyuan. Therefore, in this work, the epidemiology of adult female SUI in Taiyuan was investigated and analyzed. It can not only understand the prevalence of SUI and related influencing factors in Taiyuan area, but also enrich the epidemiological data of SUI in Taiyuan area, lay a foundation for epidemiological investigation in China, and is more conductive to establishing our own prevention standards with reference to international standards combined with China's national conditions, so that they can achieve the ultimate purpose of early understanding, early detection, early diagnosis and treatment and reducing the prevalence.

## Materials and methods

### Study participants and criteria

Adult female aged 18 years and older were randomly selected in the Taiyuan area. Inclusion criteria: (1) Respondents with complete information on the questionnaire; (2) female aged 18 years and older; (3) Household registration in Taiyuan or permanent residence in the local area for more than 2 years; (4) Informed consent to participate in the survey voluntarily. Exclusion criteria: (1) respondents with mental and neurological diseases (Patients with associated diseases were excluded by viewing the population health records of the communities surveyed); (2) respondents who do not sign the informed consent; (3) respondents with incomplete questionnaire data.

### Survey period and method

The survey was conducted from February 2019 to January 2020. The questionnaire was filled in online by on-site scanning code, and a questionnaire survey was conducted using a random multilevel cluster sampling. Six districts in Taiyuan City were selected: Yingze District, Xinghualing District, Wanbailin District, Jiancaoping District, Jinyuan District, and Xiaodian District. According to the data of the National Bureau of Statistics in the past two years, under the condition of ensuring the balance of economic level, living environment, and occupational distribution of the sampled survey sites, each part was then randomly selected from a sub-district and township, and then a community and a village were selected from each sub-district and township according to the random cluster sampling, with a total of 12 areas. Estimation of sample size: The number of investigators is calculated according to the epidemiological formula n = 400 (1 − P)/P (P is the estimated prevalence). It has been reported [8] that the average prevalence of SUI is 27.5% in urban areas and 32.5% in rural areas in China, based on which it is estimated that the sample size should be greater than 1055 in urban areas and greater than 831 in rural areas. The prevalence was measured as a percentage of the total number of patients with a symptom. This survey is an anonymous survey. A specially-assigned person explains the significance of this survey to the investigators and provides free consultation service. The survey is voluntarily filled in to ensure the quality of the survey. All female included in the study gave informed consent and signed an informed consent form. This study was approved by the Ethics Committee of First Hospital of Shanxi Medical University.

### The content of the questionnaire

The questionnaire (Additional file: 1) was designed mainly based on the ICIQ-FLUTS Questionnaire and it also took into account demographic and socioeconomic characteristics of female in Taiyuan, and the survey on the willingness to seek medical treatment for SUI and the way to obtain SUI-related knowledge was also added. The questionnaire contained relevant questions on demographic characteristics (such as place of residence (urban and rural), age, occupation, body mass index (BMI), physical exercise, sedentary, smoking, drinking, daily water intake, diet), obstetrics and gynecology (such as age at menarche, sexual history, oral contraceptives, number of miscarriages, age at first birth, number of deliveries, number of pregnancies, mode of delivery, birth weight, episiotomy, dystocia, menopause), and other factors (such as a history of pelvic surgery, urinary tract infection, defecation, holding urine, making the bladder empty faster by pushing down). Among them, mode of delivery and birth weight refer to the first delivery.

### Statistical analysis

Statistical software SPSS 22.0 was used for data entry and statistical analysis. Expression of enumeration data by rate, univariate analysis of risk factors for SUI was performed using the Chi-square (χ^2^). Multivariate logistic regression analysis was performed on data that were statistically significant after univariate analysis, categorical variables with the first group as dummy variables. *P* < 0.05 was considered statistically significant.

## Results

A total of 4352 questionnaires were sent out in this survey, and 4004 valid questionnaires were returned, with a recovery rate of 92%. Of the 4004 adult female, 1341 were diagnosed with SUI. The prevalence of SUI was 33.5%.

### Univariate analysis of risk factors for SUI

#### Relationship between demographic data and SUI

This survey showed that there were statistically significant difference (*P* < 0.05) between the SUI and non-SUI groups in the comparison of the place of residence (urban and rural), age, occupation, body mass index (BMI), sedentary, smoking, drinking, daily water intake and diet, while there was no statistically significant (*P* > 0.05) difference in the comparison of whether physical exercise was performed (Table 1).

**Table 1:** Relationship between demographic characteristics and SUI

Demographic characteristics	N	SUI		χ^2^	*P*
		N	%		
Place of residence					
Urban	1869	564	30.2	17	< 0.001
Rural	2135	777	36.4		
Age (years)					
20–29	1455	333	22.9	128	< 0.001
30–39	820	293	35.7		
40–49	882	384	43.5		
50–59	532	201	37.8		
≥ 60	315	130	41.3		
Occupation					
Manual work	637	279	43.8	55	< 0.001
Mental work	1944	563	29		
Manual/mental work	783	295	37.7		
Unemployed	640	204	31.9		
BMI (kg/m^2^)					
< 18.5	464	130	28	154	< 0.001
18.5–24	2353	644	27.4		
> 24	1187	567	47.8		
Physical exercise (times/week)					
0	1784	620	34.8	6.6	0.09
1–2	1183	401	33.9		
3–4	595	173	29.1		
≥ 5	442	147	33.3		
Sedentary					
Yes	2537	764	30.1	35	< 0.001
No	1467	577	39.3		
Smoking					
Yes	92	45	48.9	10	0.002
No	3912	1296	33.1		
Drinking					
Yes	217	56	25.8	6	0.01
No	3787	1285	33.9		
Daily water intake (ml)					
< 500	533	150	28.1	8	0.04
500–1000	2212	753	34		
1000–2000	927	324	35		
> 2000	332	114	34.3		
Diet					
Mainly staple food (noodles, buns)	1775	690	38.9	55	< 0.001
Mainly vegetables and fruits	221	40	18.1		
Mainly meat protein	87	29	33.3		
Balanced diet consisting of three types above	1921	582	30.3		

*SUI* stress urinary incontinence, *BMI* body mass index

#### Relationship between obstetrical and gynecological factors and SUI

There were statistically significant difference (*P* < 0.05) between the SUI and non-SUI groups in the comparison of the obstetrical and gynecological factors (such as age at menarche, sexual history, oral contraceptives, number of pregnancies, number of miscarriages, age at first birth, mode of delivery, birth weight, number of deliveries, episiotomy, dystocia, menopause) (Table 2).

**Table 2:** Relationship between obstetrical and gynecological factors and SUI

Relevant factors	N	SUI		χ^2^	*P*
		N	%		
Age at menarche					
11	191	42	22	15	0.001
12–15	2794	973	34.8		
> 15	1019	326	32		
Sexual history					
Yes	3145	1178	37.5	103	< 0.001
No	859	163	19		
Oral contraceptives					
Yes	854	328	38.4	12	0.001
No	3150	1013	32.2		
Number of pregnancies					
0	1222	212	17.3	221	< 0.001
1–2	1652	623	37.7		
≥ 3	1130	506	44.8		
Number of miscarriages					
0	2450	710	29.5	55	< 0.001
1–2	1389	571	41.1		
≥ 3	210	60	28.6		
Age at first birth					
< 20	78	33	42.3	211	< 0.001
20–25	1409	569	40.4		
26–30	1170	492	42.1		
> 30	103	22	21.4		
Mode of delivery					
Vaginal	2038	906	44.5	235	< 0.001
Cesarean section	693	187	27		
Birth weight (kg)					
< 3.5	1692	694	41	184	< 0.001
≥ 3.5	1040	409	39.3		
Number of deliveries					
0	1828	390	21.3	227	< 0.001
1–2	1764	754	42.7		
≥ 3	412	197	47.8		
Episiotomy					
Yes	594	320	53.9	233	< 0.001
No	2169	784	36.1		
Dystocia					
Yes	201	106	52.7	190	< 0.001
No	2570	1004	39.1		
Menopause					
Yes	1040	394	37.9	12	< 0.001
No	2964	947	32		

*SUI* stress urinary incontinence

#### Relationship between other factors and SUI

Except for the history of pelvic surgery, other factors (urinary tract infection, defecation, holding urine, making the bladder empty faster by pushing down) were related to the prevalence of SUI (*P* < 0.05) (Table 3).

**Table 3:** Relationship between other factors and SUI

Other factors	N	SUI		χ^2^	*P*
		N	%		
History of pelvic surgery					
Yes	246	95	38.6	3.1	0.08
No	3758	1246	33.2		
Urinary tract infection					
Yes	1082	480	44.4	79	< 0.001
No	2922	861	29.5		
Holding urine					
Yes	2042	792	38.8	52	< 0.001
No	1962	549	28		
Defecation					
Smooth	3107	971	31.3	32	< 0.001
Force	718	301	41.9		
Very laborious	179	69	38.5		
Making the bladder empty faster by pushing down					
Yes	3393	1093	32.2	16	< 0.001
No	611	248	40.6		

*SUI* stress urinary incontinence

### Logistic multivariate regression analysis of risk factors for SUI

Multivariate logistic regression analysis was performed with variables with *P* < 0.05 in univariate analysis as independent variables and presence or absence of SUI (no = 0, yes = 1) as dependent variables, and the results showed that: compared with female with BMI < 18.5 kg/m^2^, those with BMI 18.5–24 kg/m^2^ (OR = 1.6, 95% CI = 1.2–2.1, *P* = 0.001) and those with BMI > 24 kg/m^2^ (OR = 2.4, 95% CI = 2.0–2.8, *P* < 0.001) had a higher prevalence of SUI; compared with the dietbased on staple foods, the diet based on vegetables and fruits (OR = 0.25, 95% CI = 0.16–0.41, *P* < 0.001), the diet based on meat and protein (OR = 0.91, 95% CI = 0.84–0.99, *P* = 0.02), and balanced diet consisting of three types above (OR = 0.31, 95% CI = 0.15–0.62, *P* = 0.001) could reduce the occurrence of SUI; female who delivered 1–2 times had a 1.3 times risk of SUI than those who did not (OR = 1.3, 95% CI = 1.0–1.5, *P* = 0.02), those who delivered ≥ 3 times had a 2.6 times risk of SUI than those who did not (OR = 2.6, 95% CI = 1.8–3.8, *P* < 0.001). In addition, place of residence (OR = 1.2, 95% CI = 1.0–1.5, *P* = 0.03), smoking (OR = 1.8, 95% CI = 1.1–3.0, *P* = 0.02), mode of delivery (OR = 2.0, 95% CI = 1.6–2.6, *P* < 0.001), dystocia (OR = 1.3, 95% CI = 1.1–1.6, *P* < 0.001), menopause (OR = 1.4, 95% CI = 1.1–1.8, *P* = 0.004), oral contraceptives (OR = 1.3, 95% CI = 1.1–1.5, *P* = 0.01), urinary tract infections (OR = 1.4, 95% CI = 1.2–1.7, *P* < 0.001), making the bladder empty faster by pushing down (OR = 1.5, 95% CI = 1.2–1.9, *P* < 0.001), and holding urine (OR = 1.7, 95% CI = 1.4–1.9, *P* < 0.001), were found to be risk factors for adult female SUI in Taiyuan (*P* < 0.05) (Table 4).

**Table 4:** Logistic multivariate regression analysis of risk factors for SUI

Risk factors	β	SE	Wald	*P*	OR	95% CI
Place of residence	0.21	0.09	5.1	0.03	1.2	1.0–1.5
Smoking	0.62	0.25	5.8	0.02	1.8	1.1–3.0
BMI (kg/m^2^)			105	< 0.001		
18.5–24	0.46	0.14	11	0.001	1.6	1.2–2.1
> 24	0.87	0.09	103	< 0.001	2.4	2.0–2.8
Age (years)			4.7	0.32		
30–39	0.09	0.15	0.4	0.54	1.1	0.82–1.5
40–49	− 0.12	0.15	0.6	0.43	0.89	0.67–1.2
50–59	0.12	0.21	0.3	0.55	1.1	0.75–1.7
≥ 60	0.13	0.23	0.3	0.57	1.1	0.72–1.8
Diet			45	< 0.001		
Mainly vegetables and fruits	− 1.4	0.25	32	< 0.001	0.25	0.16–0.41
Mainly meat protein	− 0.09	0.04	5.5	0.02	0.91	0.84–0.99
Balanced diet consisting of three types above	− 1.2	0.36	11	0.001	0.31	0.15–0.62
Sexual history	0.12	0.15	0.6	0.44	1.1	0.84–1.5
Number of deliveries			24	< 0.001		
1–2	0.23	0.1	5.4	0.02	1.3	1.0–1.5
≥ 3	0.96	0.2	24	< 0.001	2.6	1.8–3.8
Mode of delivery	0.72	0.13	32	< 0.001	2	1.6–2.6
Dystocia	0.28	0.08	13	< 0.001	1.3	1.1–1.6
Menopause	0.36	0.12	8.3	0.004	1.4	1.1–1.8
Oral contraceptives	0.25	0.1	6.3	0.01	1.3	1.1–1.5
Urinary tract infection	0.35	0.09	15	< 0.001	1.4	1.2–1.7
Making the bladder empty faster by pushing down	0.42	0.11	15	< 0.001	1.5	1.2–1.9
Holding urine	0.51	0.08	41	< 0.001	1.700	1.4–1.9

*SUI* stress urinary incontinence, *BMI* body mass index, *SE* standard error, *CI* confidence interval, *OR* odds ratio

### The willingness to seek medical treatment of SUI

**Fig. 1:**
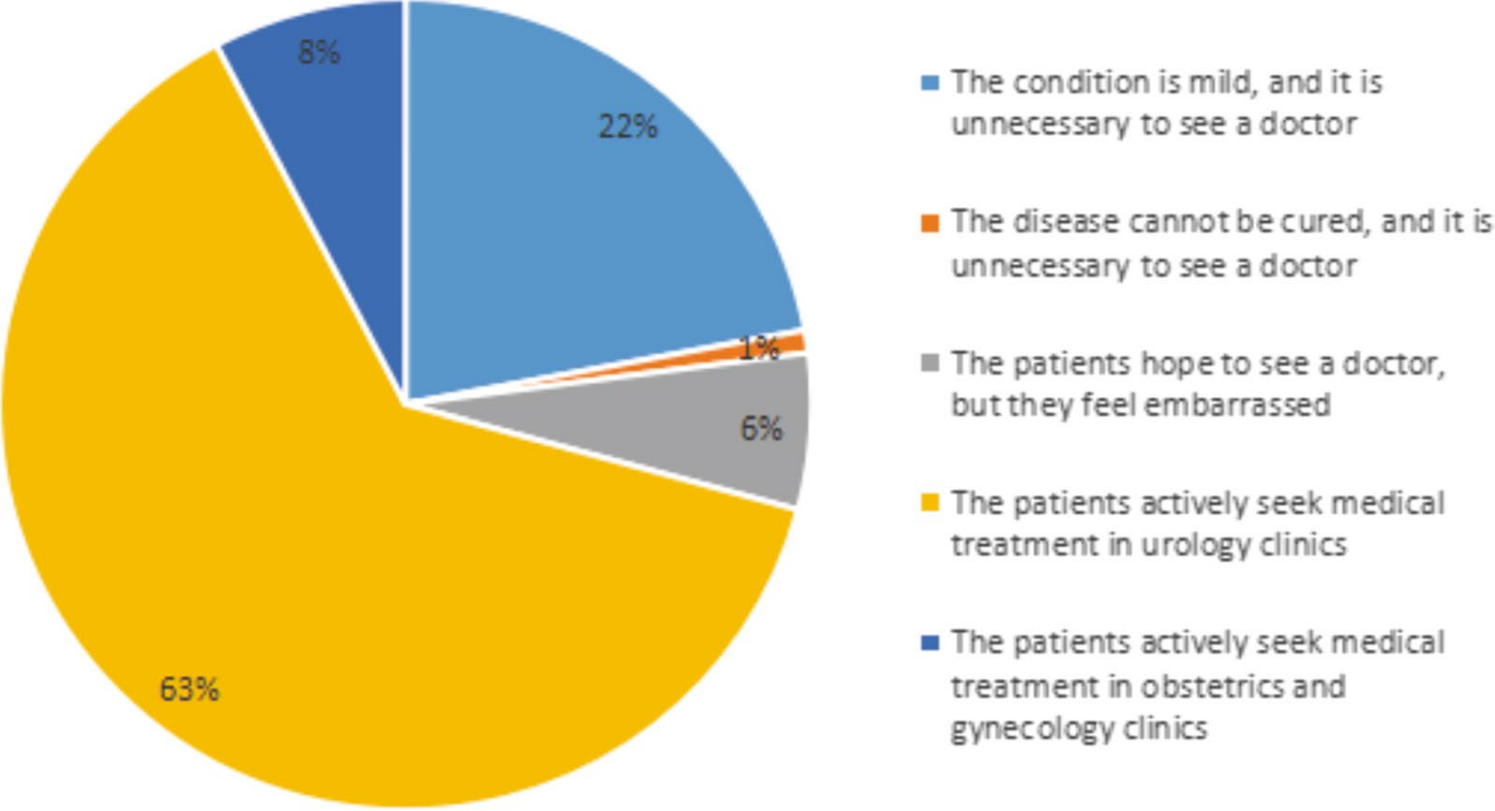
The willingness to seek medical treatment of SUI

The willingness of patients to seek medical treatment was also surveyed, as shown in Fig. 1. The results show that among the 4004 adult females surveyed, 22% and 1% believed that the disease was mild and incurable, respectively, without need to seek medical attention; 6% were embarrassed to seek medical treatment; 63% and 8% believed that they should be actively treated by urologists and Obstetrics and Gynecology (OB/GYN) doctors, respectively.

### Ways for respondents to acquire SUI-related knowledge

**Fig. 2:**
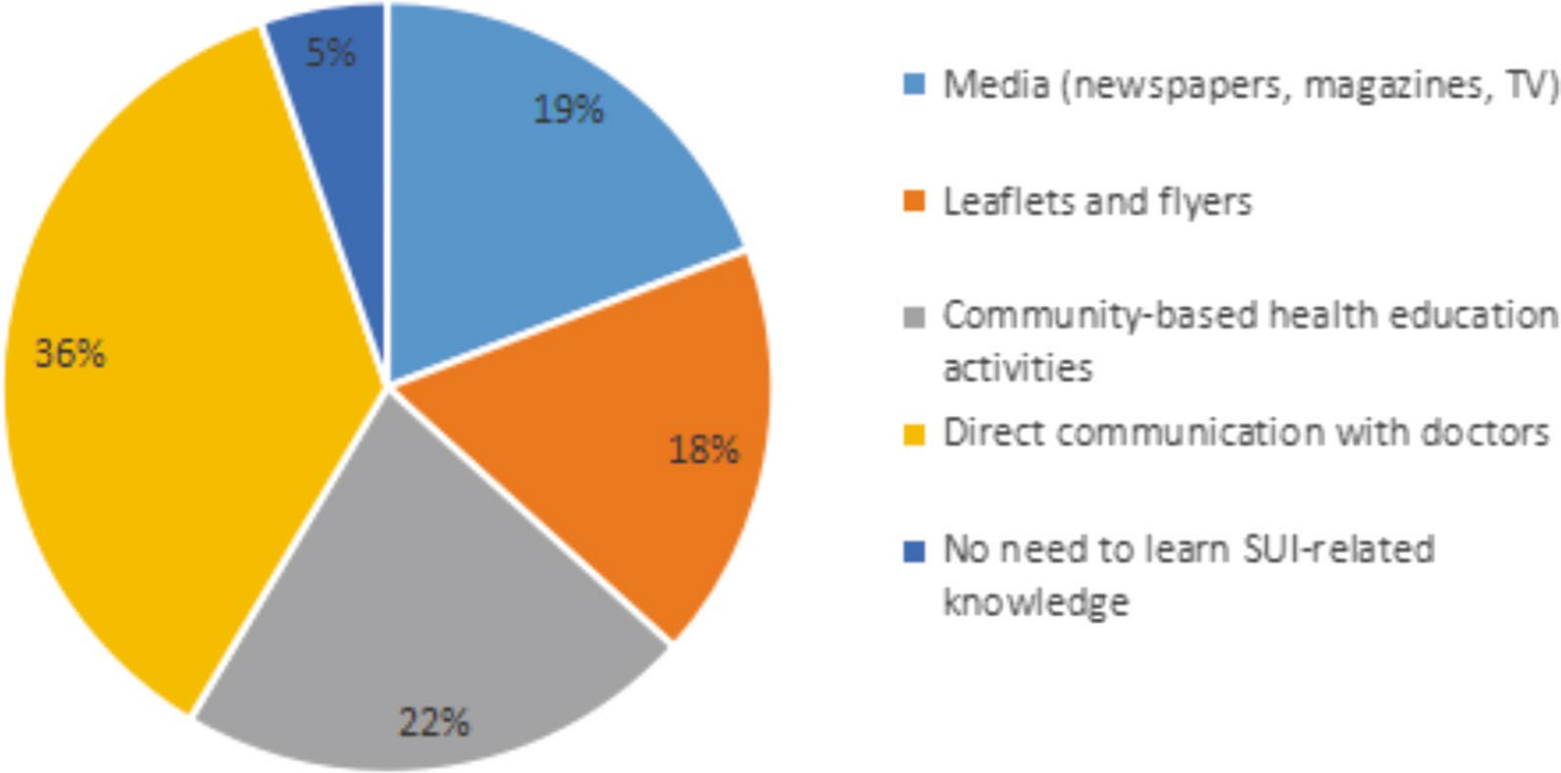
Ways for respondents to acquire SUI-related knowledge

The results show that the respondents obtained SUI-related knowledge through media (19%), leaflets (18%), community education activities (22%), and direct communication with doctors (36%); only 5% believed that they did not need to understand SUI-related knowledge (Fig. 2).

## Discussion

### Prevalence of female SUI

As the population ages, SUI is more common in female and exerts a substantial burden on patients, their families, and society [9]. At present, there are many discussions on SUI risk factors, and there is no uniform conclusion, of which the highest discussion is related to age and delivery [10–13]. Due to the differences in regional environments, dietary habits, and lifestyles, the prevalence of SUI in China varies in different regions. Therefore, risk factors for SUI might also differ in different regions. Epidemiological survey shows that the prevalence of SUI in adult female is about 18.9% in China, while Wu et al. [14] reported the prevalence of SUI in adult female is 2.3–45.8% in other countries. Yu et al. [8] found that SUI was the main subtype of urinary incontinence in adult female in China, with an average prevalence of 27.5% in urban areas and 32.5% in rural areas, as well as 30.9% in southern China and 31.4% in northern China. Similarly, we found that the overall prevalence of SUI in adult female in Taiyuan was 33.5%, with 30.2% in urban areas and 36.4% in rural areas.

### Risk factors for SUI

#### Demographic characteristics and SUI

Previous reports [15–17] have shown that SUI increases with age. However, this survey found that SUI was not correlated with age in Taiyuan (*P* > 0.05) (Table 4), and the highest prevalence of SUI was found in female aged 40–49 years, possibly due to changes in hormone levels. In addition, it is found that the prevalence of SUI in young female aged 20–29 years was 22.9%, which was similar to the results presented by Zhou et al. [18], indicating that the prevalence of SUI is increasing in young female.

The present survey found that place of residence was one of the important risk factors for SUI. The reasons may be that female in rural areas are more engaged in manual labor than female in urban areas. Manual labor is more likely to increase abdominal pressure and cause damage to pelvic floor muscle and ligament, thereby increasing the prevalence of SUI [19]. In addition, the prevalence of SUI was also closely associated with smoking, BMI, and diet. It is reported [20, 21] that the relative risk of SUI in current smokers is between 1.8 and 2.92. Either by direct effect or indirectly through smoking-related illnesses that cause increased coughing, smoking seems to have a strong causal relationship with SUI [22]. Smoking may affect collagen synthesis and cause severe cough, which may exacerbate the anatomical defects and affect the pressure transmission of abdominal pressure to the urethra, thereby exerting more pressure on the bladder. Based on BMI, the respondents surveyed were divided into three groups: underweight (BMI < 18.5 kg/m^2^), normal weight (BMI 18.5–24 kg/m^2^) and overweight (BMI > 24 kg/m^2^). As shown in Table 1, the multivariate logistic regression analysis showed that the prevalence of SUI significantly increased with the increasing BMI (*P* < 0.05). Possibly owing to the increased body weight, the abdominal weight, intra-abdominal pressure, intravesicular pressure, and urethral mobility increased, leading to urinary incontinence [23]. Taiyuan belongs to the Central Plains region. The diet in Taiyuan consists mainly of starchy food, such as noodles and steamed bread. Long-term dietary intake of starchy food, only with a limited intake of high-fiber food, could increase the risk of constipation. In patients with constipation, the high abdominal pressure could cause the uterus, bladder, and anterior wall of the vagina to move down, leading to abnormal closure of the urethra and SUI [24, 25].

#### Obstetrical and gynecological factors and SUI

Some obstetrical and gynecological factors have a great impact on the prevalence of SUI (Table 4). The present results showed that the prevalence of SUI was significantly higher in female who underwent vaginal delivery than in female who underwent cesarean section, and the prevalence of SUI significantly increased with the increasing number of vaginal deliveries (*P* < 0.05). Similarly, Tähtinen et al., found that vaginal delivery increased the risk of SUI by nearly two times compared with cesarean section, with an absolute increase of 8% [26]. Dystocia was also found to be one of the risk factors for SUI. During delivery, dystocia could cause pelvic floor neuromuscular injury [27], myofascial rupture and lengthening, and the changes in the anatomical position of the bladder and urethra, which affected the ability of urethra to close and led to voiding dysfunction in female. Furthermore, menopause is an important risk factor for SUI in female. A recent study found that urinary incontinence in postmenopausal female occurs more often than other civilization diseases, such as hypertension, diabetes and depression [28]. Both urethra and vagina originate from the genitourinary sinus. Estrogen receptors are distributed in the urethral and vaginal epithelium. Estrogen is an important hormone to promote maturation of the urethral and vaginal epithelium, thereby increasing urethral closure pressure and the length of the urethra [29]. During menopause, estrogen deficiency leads to atrophy of the urethral mucosa [30]. At the same time, estrogen deficiency also affects the synthesis of collagen fibers, which are the main components of supportive tissues of the pelvic floor. Compositional changes of collagen could weaken supportive tissues of the pelvic organs, thereby increasing the risk of SUI [31]. In addition, Oral contraceptives interfere with the hypothalamic-pituitary-ovarian axis and inhibit the synthesis and release of follicle-stimulating hormone and luteinizing hormone, which causes luteal dysfunction, followed by decreased estrogen secretion [32, 33]. Estrogen and progesterone receptors have been found throughout the lower urinary tract, and many of the tissues involved in female continence have been found to be estrogen-sensitive [34]. Deficiency of estrogen weakens pelvic floor tissue support, atrophies the urethral mucosa, eventually relaxes the urethra [30, 31], and develops SUI.

#### Other factors and SUI

Patients with urinary tract infections often have storage and voiding symptoms, such as frequent urination, urgency, and feeling of incomplete bladder emptying. The common urinary incontinence is mainly urgency UI or mixed UI [19]. In this work, the logistic multivariate regression analysis showed that SUI was significantly associated with urinary tract infection (*P* < 0.05), but the mechanism of SUI remains unknown. Some female like to strain abdominal muscles and pelvic floor muscles to empty bladder fast. Though an increase in bladder pressure by abdominal straining could make urination fast and reduce the time of urination, the long-term abdominal straining to void may impair the function of detrusor in female, consequently leading to urinary incontinence [35]. It is reported that, without the initiation of the micturition reflex, the use of abdominal straining to void is more likely to cause voiding dysfunction and stress urinary incontinence [36]. Studies have found that female who often hold their urine are more likely to have urinary incontinence [37, 38]. Holding urine for too long can lead to urine leakage if the intravesical pressure exceeds urethral resistance. Furthermore, frequent holding can induce detrusor overactivity, decrease bladder compliance and contractility, and disrupt the physiological state of low-pressure filling and low-pressure voiding, resulting in a significant increase in detrusor pressure during bladder filling [39, 40] and involuntary urine leakage when the bladder pressure is higher than urethral resistance.

### The present situation and preventive measures against SUI in adult female in Taiyuan

Our survey found that adult female in Taiyuan have a higher willingness to seek medical treatment than those in other regions, with 63% of female willing to visit the urology clinics. In addition, 36% of female hope to obtain the SUI related knowledge directly from doctors, indicating the increasing awareness of SUI among adult female in Taiyuan.

SUI can be caused by multiple factors. This study identified various risk factors for adult female SUI in Taiyuan, including found that place of residence, smoking, BMI, diet, number of deliveries, mode of delivery, dystocia, menopause, oral contraceptives, urinary tract infection, making the bladder empty faster by pushing down, and holding urine. To reduce the occurrence of SUI, we propose some preventive measures as follows: alcohol withdrawal, balanced diet, weight control, healthy living habits, frequent changes of underwear, personal hygiene, timely voiding, and no straining during urination. Because the prevalence of SUI in rural areas is significantly higher than that in urban areas, the awareness and education about bladder health should be strengthened among adult female in rural areas. In addition, it is necessary to strengthen family planning publicity and education, so that more women accept the placement of intrauterine contraceptive rings and advocate condom birth control. Try to avoid oral contraceptives, maintain normal physiological estrogen and progesterone levels, thereby reducing the prevalence of female SUI. Medical professionals should provide prenatal care and tests for expectant mothers and adopt appropriate delivery methods to reduce the risk of dystocia. After delivery, female are encouraged to receive postpartum care and to do pelvic floor muscle exercises as soon as possible to increase the flexibility of pelvic floor muscles. Providing health education to perimenopausal female is also an effective way to prevent SUI.

The strength of this study are that it was a large sample survey, while the questionnaire design incorporates local characteristics in Taiyuan and adds risk factors associated with this region. However, there are still some possible limitations in our study: although our samples has more than 4,000, the samples are still small compared with the population of more than 4 million in Taiyuan, and more samples, multi-level clinical epidemiological surveys are needed. Second, our study is more representative of Taiyuan region, which may differ from the findings in other regions of China, with some regional limitations.

## Conclusion

This study conducted a large-scale epidemiological survey of SUI in adult female in Taiyuan, and the result showed that the prevalence of SUI was high. Therefore, Local health authorities should strengthen the screening of female at high risk for SUI and take effective preventive measures to reduce the prevalence of SUI.

## Supplementary Information

**Additional file 1:** Adult female stress urinary incontinence questionnaire in Taiyuan.

## Data Availability

The data used to support the findings of this study are included within the article.
