# Supplementary material for: Epidemiological survey of adult female stress urinary incontinence
Source: BMC Womens Health. 2021 Apr 22;21:172. doi: 10.1186/s12905-021-01319-z (PMC8061196; doi:10.1186/s12905-021-01319-z)
Supplement: Supplementary file 1 — Additional file 1: Adult female stress urinary incontinence questionnaire in Taiyuan. [file 12905_2021_1319_MOESM1_ESM.pdf]

Adult Female Stress Urinary Incontinence Questionnaire in Taiyuan

Dear Residents：

This questionnaire is an anonymous survey. We will keep your answers strictly confidential. Please feel free to answer your questions based on facts. Thank you for your cooperation and support.

1.Age (years) ①20~29 ②30~39 ③40~49 ④50~59 ⑤≥60

2.Height：___ m

3.Weight：___ kg

4.Place of residence ①Urban ②Rural

5.Occupation ①Manual work ②Mental work ③Manual/mental work ④Unemployed

6.Daily working hours ①No ②＜8h ③8h~12h ④＞12h

7.Physical exercise(times/week) ①0 ②1~2 ③3~4 ④≥5

8.Sedentary ①Yes ②No

9.Smoking ①Yes ②No

10.Drinking ①Yes ②No

11.Daily water intake (ml) ①＜500 ②500~1000 ③1000~2000 ④＞2000

12.Diet ①Mainly staple food (noodles, buns) ②Mainly vegetables and fruits
③Mainly meat and protein ④Balanced diet consisting of three types above

13.Age at menarche(years) ①11 ②12~15 ③＞15

14.Sexual history ①Yes ②No

15.Oral contraceptives ①Yes ②No

16.Age at first birth(years) ①＜20 ②20~25 ③26~30 ④＞30

17.Number of pregnancies(times) ①0 ②1~2 ③≥3

18.Number of miscarriages(times) ①0 ②1~2 ③≥3

19.Mode of delivery ①vaginal ②cesarean section

20.Number of deliveries(times) ①0 ②1~2 ③≥3

21.Episiotomy ①Yes ②No

22.Dystocia ①Yes ②No

23.Birth weight (kg) ①＜3.5 ②≥3.5

24.Menopause ①Yes ②No

25.Start of postpartum work(months) ①＜3 ②3~6 ③6~12 ④＞12

26.History of pelvic surgery ①Yes ②No

27.Urinary tract infection ①Yes ②No

28.Holding urine ①Yes ②No

29.Defecation ①Smooth ②Force ③Very laborious

30.Constipation ①Yes ②No

31.Making the bladder empty faster by pushing down ①Yes ②No

32.Voiding condition ①Frequency ②Urgency ③Terminal dribble
④Urination disorder ⑤Painful urination

33.Nocturia(times) ①0 ②1~2 ③≥3

34.Urinary Incontinence ①No ②Occasional ③Always

35.When do you experience urinary incontinence?
①No ②Obvious cause ③Holding urine ④Tense time
⑤Sporting activities or on sneezing or coughing

36. Are you willing to learn about urinary incontinence?

①Yes ②No

37. Do you think it is necessary to seek medical treatment of stress urinary incontinence?

①The condition is mild, and it is unnecessary to see a doctor

②The disease cannot be cured, and it is unnecessary to see a doctor

③Hope to see a doctor, but feel embarrassed

④Seek medical treatment actively in urology clinics

⑤Seek medical treatment actively in obstetrics and gynecology clinics

38. How do you want to learn about stress urinary incontinence?

①Media (newspapers, magazines, TV)

②Leaflets and flyers

③Community-based health education activities

④Direct communication with doctors

⑤No need to learn SUI-related knowledge
